# Machine learning-based detection of cardiovascular disease using ECG signals: performance vs. complexity

**DOI:** 10.3389/fcvm.2023.1229743

**Published:** 2023-07-31

**Authors:** Huy Pham, Konstantin Egorov, Alexey Kazakov, Semen Budennyy

**Affiliations:** ^1^ Department of Computer Science, HSE University, Moscow, Russia; ^2^ AI for Medicine, Sber AI Lab, Moscow, Russia; ^3^ Applied Research Center, Sber AI Lab, Moscow, Russia; ^4^ New Materials Discovery Group, Artificial Intelligence Research Institute (AIRI), Moscow, Russia

**Keywords:** ECG, cardiovascular disease, arrhythmia, deep learning, Poincaré diagram

## Abstract

**Introduction:**

Cardiovascular disease remains a significant problem in modern society. Among non-invasive techniques, the electrocardiogram (ECG) is one of the most reliable methods for detecting cardiac abnormalities. However, ECG interpretation requires expert knowledge and it is time-consuming. Developing a novel method to detect the disease early improves the quality and efficiency of medical care.

**Methods:**

The paper presents various modern approaches for classifying cardiac diseases from ECG recordings. The first approach suggests the Poincaré representation of ECG signal and deep-learning-based image classifiers. Additionally, the raw signals were processed with the one-dimensional convolutional model while the XGBoost model was facilitated to predict based on the time-series features.

**Results:**

The Poincaré-based methods showed decent performance in predicting AF (atrial fibrillation) but not other types of arrhythmia. XGBoost model gave an acceptable performance in long-term data but had a long inference time due to highly-consuming calculations within the pre-processing phase. Finally, the 1D convolutional model, specifically the 1D ResNet, showed the best results in both studied CinC 2017 and CinC 2020 datasets, reaching the F1 score of 85% and 71%, respectively, and they were superior to the first-ranking solution of each challenge. The 1D models also presented high specificity. Additionally, our paper investigated efficiency metrics including power consumption and equivalent CO_2_ emissions, with one-dimensional models like 1D CNN and 1D ResNet being the most energy efficient. Model interpretation analysis showed that the DenseNet detected AF using heart rate variability while the 1D ResNet assessed the AF patterns in raw ECG signals.

**Discussion:**

Despite the under-performed results, the Poincaré diagrams are still worth studying further because of the accessibility and inexpensive procedure. In the 1D convolutional models, the residual connections are useful to keep the model simple but not decrease the performance. Our approach in power measurement and model interpretation helped understand the numerical complexity and mechanism behind the model decision.

## 1. Introduction

Cardiovascular disease is the serious public health problem that affects millions of people worldwide and is also a leading cause of death (1). The expense of healthcare, lost productivity, and a diminished quality of life due to heart illness has a significant economic and social impact on individuals, families, and the society as a whole (2). This emphasizes the value of early disease identification. While the electrocardiogram (ECG) is considered the most crucial method for detecting and diagnosing cardiac problems (3), it takes time and requires trained professionals with specialized skills to interpret ECGs. Furthermore, the availability of devices that can record ECG signals is increasing exponentially. Nowadays, many types of wearable devices are able to collect these signals. However, the number of research studies on this type of data is limited.

The ECG analysis task includes beat annotation and signal classification. While the former deals with aligning the signal segment to the heart contraction, the latter tries to predict the disease from the signal data.

In the domain of ECG classification, there are a number of methods ranging from feature-based models to deep learning–based ones. In the feature-based model, the most common features are the domain-dependent features, statistical descriptors, morphological characteristics, and frequency-based features (4). Meanwhile, the deep learning models are also diverse. The work by Jun et al. (5) took advantage of AlexNet, VGGNet, and their customized CNN architect to predict arrhythmia diseases based on 128×128 grayscale images of ECG signal. The best model reached 0.989 Area under the ROC Curve (AUC) and over 99% accuracy. Two years later, there was a concerted effort by Hong et al. (6) to develop an ensemble system to process the waveform data. With several types of CNN-based deep learning models, this system scored an accuracy of 95%. Also, Ribeiro et al. (7) used 1D ResNet to predict six types of cardiovascular disease and got an F1 score of over 80%. After that, the work by Zhang et al. (8) proved the dominant results of deep learning compared to the traditional machine learning model. Their model could classify nine subtypes of arrhythmias with an F1 score of over 80%. The impressive idea of this work is to use the SHapley Additive exPlanations (SHAP) value to explain the model output at the individual and population levels.

To improve the quality of models, the scientific community organized many challenges with large-scale datasets, including The PhysioNet/Computing in Cardiology Challenge (CinC) 2017 and 2020 (9, 10). While CinC 2017 focused on arrhythmia disease, CinC 2020 contained ECG signals in a wide range of cardiac abnormalities. There are many efforts to apply machine learning/deep learning approaches to reach the highest performance. In the challenge CinC 2017, Kamaleswaran et al. (11) used a convolutional neural network in signal processing and got the best ranking in arrhythmia prediction. After that, Natarajan et al. (12) took advantage of transformer architecture to get the highest place at challenge CinC 2020.

Besides the improvement of classification performance, the architecture of models becomes more complicated, so they require more energy to train and have a long inference time. This problem limits the application of the method, especially in handheld and wearable devices.

In this study, we focused on enhancing ECG classification approaches in terms of performance, numerical complexity, inference time, and its interpretability.

*Contribution.* The contribution of our paper is threefold:

• First, we introduce a pipeline for ECG classifier evaluation in terms of performance and numerical complexity.
• Second, we achieved a state-of-the-art level (with regard to CinC 2017 and CinC 2020 challenges) performance with the 1D ResNet model for both CinC 2017 and CinC 2020 benchmarks.
• Third, we provided interpretation techniques for Poincaré-based DenseNet121 and 1D ResNet models.

## 2. Materials and methods

### 2.1. Data

The data were collected from two challenges: PhysioNet/CinC Challenge 2017 and 2020 (9, 10). These collections were chosen because they are both open-sourced datasets with well-annotated labels, as well as massive sources of ECG recordings, which are suitable for training deep learning models. We intentionally did not apply any inclusion/exclusion criteria for data selection. The total number of disclosure data samples was split into train/validation/test subsets with the ratio 60/20/20. The partition was done by splitting randomly the dataset based on the number of recordings. In this study, we intentionally did not apply any inclusion/exclusion criteria for data selection to capture the full spectrum of patient profiles and maximize the generalizability of our findings. The details of the data are shown in Table Table 1.

**Table 1:** Descriptive statistics and demographics information of CinC 2017 and CinC 2020 datasets.

Measures	Factors	CinC 2017			CinC 2020		
		Train	Validation	Test	Train	Validation	Test
# samples		5,116	1,706	1,706	25,860	8,620	8,621
Rate (Hz)		300	300	300	257–1,000	257–1,000	257–1,000
Signal length (s)	Mean	32.4	32.8	32.5	15.4	16.1	16.1
	Min	9.1	9.8	9.0	5.0	5.0	5.0
	Median	30.0	30.0	30.0	10.0	10.0	10.0
	Max	61.0	60.6	60.8	1,800.0	1,800.0	18,00.0
Age (%)	Younger than 18	—	—	—	0.7	0.7	0.8
	18–29	—	—	—	5.4	5.2	5.2
	30–39	—	—	—	6.4	6.8	7.0
	40–49	—	—	—	11.1	11.3	10.9
	50–64	—	—	—	30.9	31.1	30.8
	65 and older	—	—	—	45.0	44.3	44.9
	Missing data	—	—	—	0.5	0.6	0.4
Gender (%)	Male	—	—	—	53.3	53.1	52.5
	Female	—	—	—	46.7	46.9	47.5
	Missing data	—	—	—	0.0	0.0	0.0

The CinC 2017 dataset was recorded by AliveCor device and contains 8,528 single-lead signals. The length of recordings is from 9 to 60 s, and the average length is about 32 s. Every ECG signal was recorded at 300 Hz and already filtered by the recorder. The host provided the data in WFDB format with a .mat file containing signal data and a .hea file containing headers for basic information including ID and recording parameters.

The CinC 2020 dataset contains 12-lead signals which come from five different sources: CPSC Database and CPSC-Extra Database, INCART Database, PTB and PTB-XL Database, The Georgia 12-lead ECG Challenge (G12EC) Database, and the Private Database.

The dataset CPSC is the collection of the ECG signals of Chinese patients, which were recorded at 500 Hz. The patient’s gender and age were disclosed in this dataset; however, the age of over-89-year-old patients is masked as 92 due to the HIPAA guidelines. The INCART database contains 30-min recordings at 257 Hz while the PTB and Georgia datasets consist of 10-s recordings only. The private part of data is not public so this source was not included in our work. The remaining dataset was split into train/test/split with the ratio 60/20/20. Like the CinC 2017 dataset, the data from CinC 2020 is also WFDB-compliant. The header files embedded the demographics information and diagnosis labels.

The embedded headers in CinC 2020 reveal the distribution of age and gender. Most of the subjects in CinC 2020 are aged 65 and older, followed by the age group of 50–64 years. The remaining age groups have relatively smaller proportions. Additionally, every subset has minimal missing data for age, with less than 1% of entries having no specified age information. Regarding gender, the sex ratio is relatively balanced. Notably, there are nearly zero missing data points for gender in CinC 2020.

**Figure 1:**
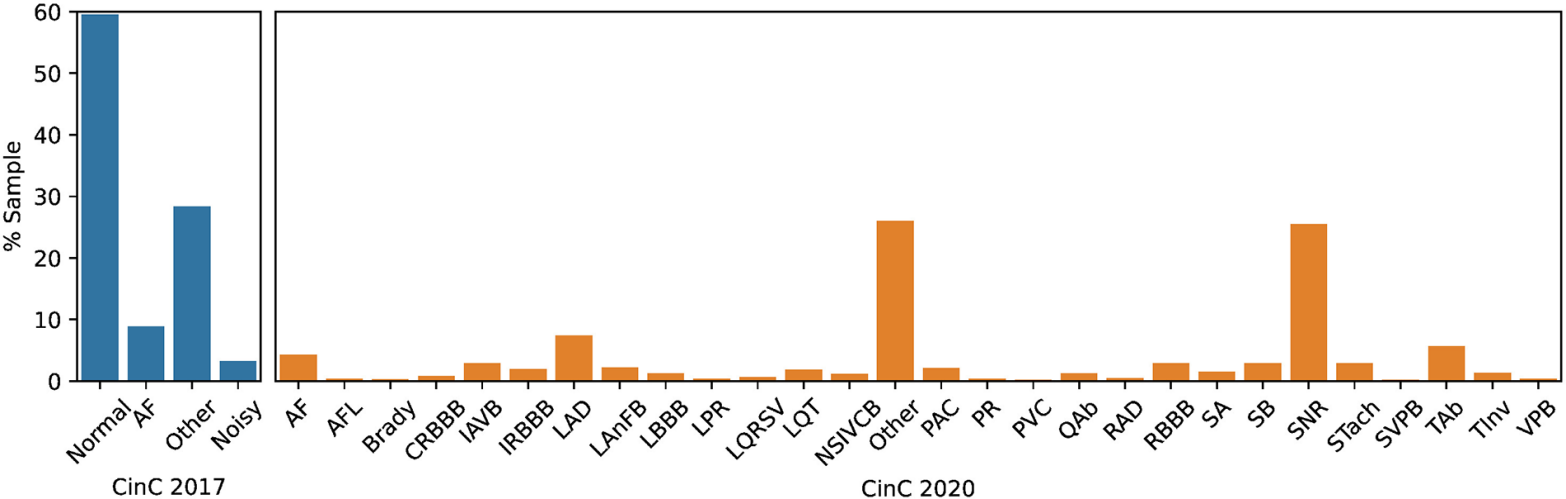
The classes distribution in datasets CinC 2017 (left side) and CinC 2020 (right side).

In term of label distribution, while the CinC 2017 dataset was limited to four classes, including Normal, Atrial Fibrillation (AF), Other, and Noisy, the CinC 2020 dataset has 28 categories of cardiovascular disease (Figure 1). The distribution of classes is very imbalanced. In CinC 2017, the AF samples are under 10% of the dataset while the Normal samples are nearly 60%. Likewise, within the CinC 2020 dataset, the Sinus Rhythm and Other categories exceed 20%, whereas the remaining classes fall below 10%. The detail description of each class is mentioned in Table S1 in the Supplementary Material.

### 2.2. Model architecture, training pipeline, and experiment conditions

**Figure 2:**
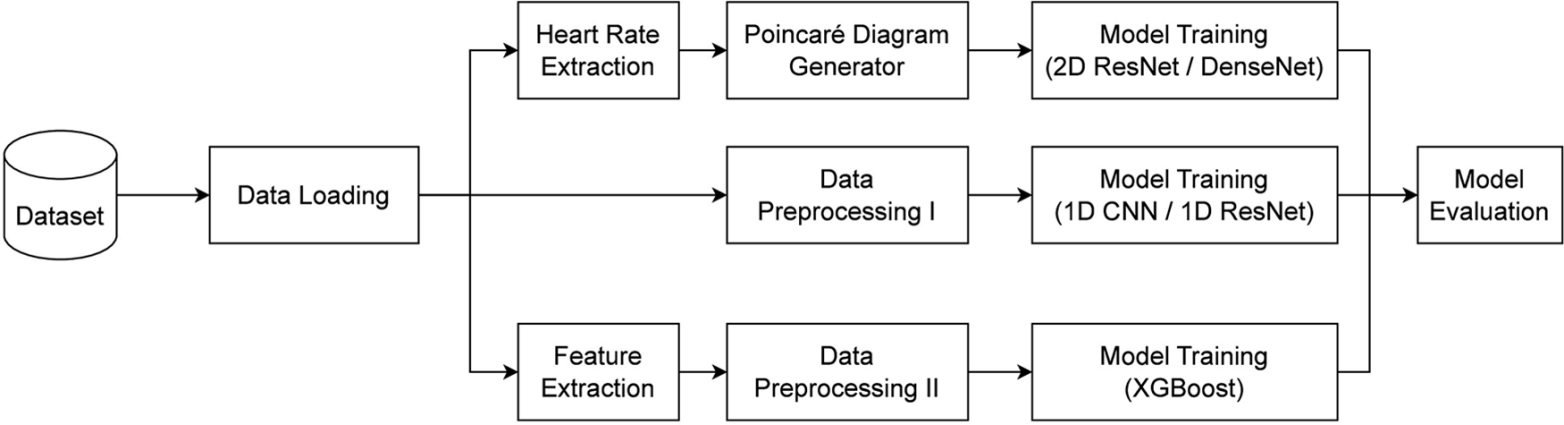
The high-level scheme of training pipeline, including data preparation, model optimization, and performance evaluation.

This section describes the details of the configuration of each model as well as the flow of data when training the model. The overview of the training pipeline is given in Figure 2 and interpreted in the following. There are three main flows of learning in our experiments. They shared the same signal loader and evaluation method. Each flow had a separate way of pre-processing data and model optimization, which will be described in detail below.

All experiments were conducted on the same computer with 1 × Intel Core i7-9700F CPU, 64 GB RAM, and 1 × NVIDIA GeForce GTX 3060 GPU. The metrics was estimated on both 5-fold cross-validation and a separated test set. In cross-validation experiments, we combined the train and validation subset into one dataset before performing the fivefold cross-validation. To estimate the performance on the unseen data, we trained the models on the train subset and tested on the separated test subset, while the validation subset was used to optimize the hyperparameters.

#### 2.2.1. Learning over Poincaré representation

**Figure 3:**
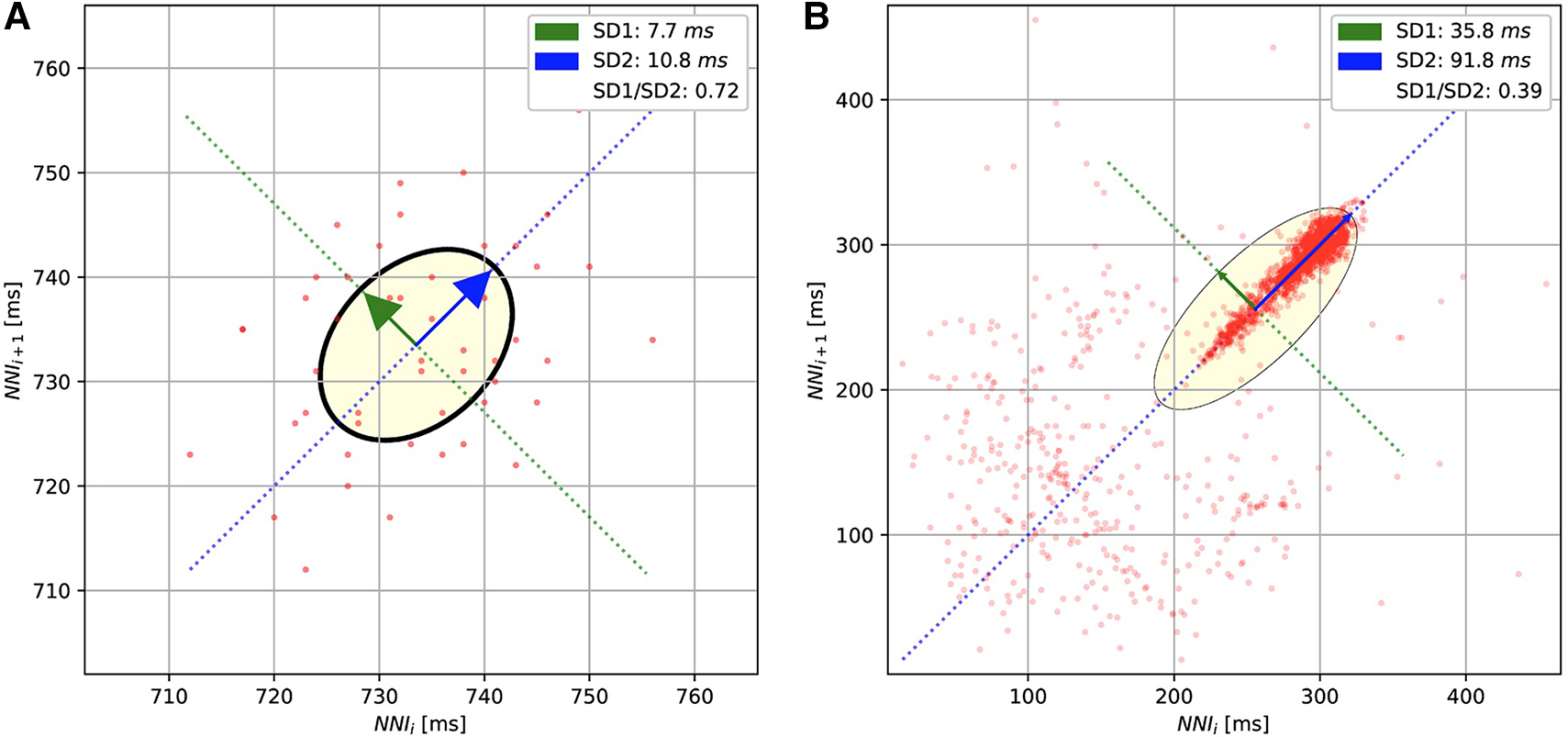
The Poincaré diagrams of the short-term (**A**) and long-term (**B**) ECGs. The diagrams plot the normal R-peak intervals (or NN intervals).

For the methods based on the Poincaré diagram, the input ECG signals were preprocessed by biosppy (13) to extract the R-peak positions from the signal (14). This library filters the ECG signal in the frequency range from 3 to 45 before using the Hamilton algorithm (15) to detect the R-peak. The distance between R-peaks (or RR intervals) was evaluated from the R-peak location. Furthermore, in our study, we only used the NN intervals, which are the distances between normal R-peaks collected after removing the noise and artifacts. The Poincaré diagram was constructed by plotting the scatter charts for $NN_{i}$ and $NN_{i+1}$ intervals. Figure 3 shows the examples of Poincaré diagram of a short and long recording.

To predict the cardiovascular disease over the Poincaré diagrams, the default architecture of ResNet50 (16) and DenseNet121 (17) were used to train from the scratch (without pre-trained weights). The last layers of these models were also tailored to match the number of classes of each dataset.

#### 2.2.2. Learning over 1D signal

In our study, the 1D CNN model comprises 12 base blocks. Each base block consists of a 1D Convolutional layer, 1D Batch Normalization, Activation function, Pooling layer, and Drop-out layer. The 1D Convolutional layer was set with no padding and stride size of 1. The output channel starts at 256 and decreases gradually to 32 in the last convolutional layer, and the kernel size starts at 20 followed by 5 layers with a kernel size of 5, and then 3 for the remaining layers. The Batch Normalization layers have the number of weights the same as the number of output channels of the prior convolutional layer. The momentum of normalization is 0.99 for every block. The Pooling of base block is Max Pooling of which the kernel size and stride size are 2. The dropout probability is set to 0.3 in every place. Before flattening the tensor and feeding to the last fully connected layer for the logit outputs, there is an average pooling layer with a kernel size of 1 and stride size of 2.

The structure of the base block in 1D ResNet includes a 1D Convolutional layer, 1D Batch Normalization, ReLU activation function, a Drop-out layer, another 1D Convolutional layer, and 1D Batch Normalization. In a base block, the input would go through these layers before adding the residual which is also the input tensor. This summation is activated by the ReLU function after leaving the block.

In our work, the 1D ResNet starts with a 1D Convolutional layer with a kernel size of 15 and the number of output channels is 64 followed by a 1D Batch Normalization, ReLU Activation function, and Max Pooling layer. After that, there are four base blocks with kernel sizes increasing from 65 to 256. The output of the last base block goes through two pooling layers: an Average Pooling layer and a Max Pooling layer. These outputs are concatenated before feeding to the final fully connected layer to compute the output logits.

In both 1D CNN and 1D ResNet models, the signals are converted to first-order difference and scaled to zero mean and unit variance before transferring to the models.

#### 2.2.3. Learning over XGBoost feature space

In the pipeline of the XGBoost model, the processed ECG signals need to feed to module tsfresh to extract the features before training model. The features extraction used the list of efficient features set but filtered out the time-consuming features including: entropy-related features, matrix profile, the number of Continuous Wavelet Transform (CWT) peak, partial autocorrelation, aggregated linear trend, and the statistics of Augmented Dickey–Fuller test. In the feature matrix, the pipeline filled the missing data with −999 and removed the low-variance features.

The hyperparameters of XGBoost were optimized by searching within the predefined space (Table 2). The optimum collection was found by Bayesian optimization implemented in the library scikit-optimize. (18) The number of search trials was limited to 100 because of time constraints.

**Table 2:** The hyperparameters searching space of XGBoost.

Component	Hyperparameter	Range	Distribution
Feature elimination	min_features_to_select	[10,\# features]	Uniform
XGBClassifier	max_depth	[2,100]	Uniform
XGBClassifier	gamma	$\left[10^{-3},10^{3}\right]$	Log-uniform
XGBClassifier	eta	$\left[10^{-3},10^{3}\right]$	Log-uniform
XGBClassifier	scale_pos_weight	$\left[10^{-3},10^{3}\right]$	Log-uniform
XGBClassifier	reg_lambda	$\left[10^{-3},10^{3}\right]$	Log-uniform
XGBClassifier	reg_alpha	$\left[10^{-3},10^{3}\right]$	Log-uniform

### 2.3. Metrics and model interpretation

The popular metrics for the classification problem include accuracy, precision, recall, and F1 score. While the accuracy is biased if the dataset is heavily imbalanced among classes (19), precision and recall are preferred in these cases. In this study, we used the harmonic mean of precision and recall (F1 score), which is easy to compare methods with only one measure. Besides the F1 score, sensitivity and specificity were also calculated to get more insight into each model. These scores were calculated as

$$\begin{array}{rl} \text{Precision} & =\frac{TP}{TP+FP},\;\text{Recall/Sensitivity}=\frac{TP}{TP+FN}\\ \text{Specificity} & =\frac{TN}{FP+TN},\;\text{F1 Score}=\frac{2\times \text{Precision}\times \text{Recall}}{\text{Precision}+\text{Recall}} \end{array}$$

where TP, FP, TN, and FN are the number of True Positive, False Positive, True Negative, and False Negative extracted from the confusion matrix, respectively. Because the dataset is multiple classes (in CinC 2017) or multiples labels (in CinC 2020), the average of each metric was reported in our study.

To assess the power efficiency of the proposed methods, we measured the power consumption, the equivalent ${\mathrm{CO}}_{2}$ emission, and the inference time of each model. The inference time was calculated by the average time of predictions on the test set. The power consumption and carbon emissions information were monitored and calculated using the open-source library *eco2AI*, which is available at https://github.com/sb-ai-lab/Eco2AI (20).

To explore the mechanism behind the decision of (1D and 2D) convolutional model, the Gradient-weighted Class Activation Mapping or GradCAM (21) was constructed to visualize which region in the input that the model focused on when predicting. The basic idea of GradCAM is to calculate the gradient of output with respect to the feature map of last convolutional layers, and then these gradients are aggregated to obtain the weight for the feature map. An heatmap is built based on these weights to show the contribution of each region to the prediction score.

For the XGBoost model, the feature importance of each feature was calculate based on the frequency of that feature in splitting data when training the tree. Because the number of variables is enormous, they were aggregated into common group.

## 3. Results

In this section, we summarize the classification performance and power consumption metrics of each classifier. After that, we try to interpret the model decision using the mentioned methods.

### 3.1. Cardiovascular diseases classification

The experiment results showed the superior performance of the 1D ResNet model learned over raw data in both datasets. Especially, in both datasets, this model surpassed the first rank solution in many measurements. The comparison of F1 scores, sensitivity, specificity, and the efficiency metrics (power consumption, equivalent ${\mathrm{CO}}_{2}$) are given in Table 3.

**Table 3:** Performance benchmark results on the fivefold cross-validation and the separated test datasets.

Dataset	Input data	Model	Fivefold cross-validation			Test set		
			F1	SENS	SPEC	F1	SENS	SPEC
CinC 2020	Poincaré	ResNet50	0.70 (0.02)	0.64 (0.03)	0.82 (0.05)	0.71	0.64	0.79
	Poincaré	DenseNet121	0.75 (0.02)	0.71 (0.02)	0.82 (0.02)	0.77	0.73	0.80
	Raw signal	1D CNN	0.81 (0.01)	0.79 (0.01)	0.86 (0.01)	0.84	0.83	**0.86**
	Raw signal	1D ResNet	**0.82** (0.01)	**0.81** (0.01)	**0.87** (0.01)	**0.85**	**0.85**	0.86
	Raw signal	Kamaleswaran et al.^a^	0.80 (0.02)	0.77 (0.02)	0.85 (0.03)	0.84	0.83	0.86
	Time series	XGBoost	0.64 (0.02)	0.72 (0.09)	0.51 (0.15)	0.69	0.82	0.48
CinC 2020	Poincaré	ResNet50	0.47 (0.03)	0.37 (0.03)	0.81 (0.03)	0.45	0.35	0.83
	Poincaré	DenseNet121	0.50 (0.00)	0.40 (0.01)	0.80 (0.01)	0.50	0.41	0.80
	Raw signal	1D CNN	0.67 (0.00)	0.59 (0.01)	0.89 (0.01)	0.69	0.63	0.88
	Raw signal	1D ResNet	**0.71** (0.00)	0.65 (0.01)	**0.92** (0.01)	**0.71**	0.65	**0.92**
	Raw signal	Natarajan et al.^a^	0.61 (0.02)	**0.82** (0.02)	0.85 (0.02)	0.66	**0.80**	0.90
	Time series	XGBoost	0.65 (0.01)	0.62 (0.03)	0.80 (0.03)	0.65	0.65	0.78

F1, F1 score; SENS, sensitivity; SPEC, specificity.

The cross-validation performance metrics are reported with mean (SD). Bold numbers indicate the performance of the best model.

^a^ The first rank solution.

For the CinC 2017 dataset, the 1D ResNet is the best-performing model with the highest F1 score in both fivefold cross-validation and separated test dataset. The model also demonstrates high sensitivity and specificity. The 1D CNN on the raw signal input also performs well among the other models. For the CinC 2020 dataset, the 1D ResNet models also outperforms other models in F1 scores and specificity. However, the sensitivity of the model is lower than the transformer-based model (12).

In detail, the Poincaré-based methods have adequate performance in the CinC 2017 challenge. However, they did not perform well in the CinC 2020 challenge. Particularly, some classes are not discriminated in the Poincaré diagrams. The models ResNet50 and DenseNet121 only identified the types Atrial fibrillation (AF), Sinus bradycardia (SB), Sinus rhythm (SNR), Sinus tachycardia (STach), and other, while the metrics for the remaining types are close to zero. This result is understandable as the information on heart rate variability is not sufficient to identify several types of heart disease.

The XGBoost is under-expected because it ranked lowest in CinC 2017 and only third in CinC 2020 despite the gradient-boosting family usually gaining the highest place at many machine learning benchmarks.

The unidimensional convolutional models yielded excellent results in both classification challenges. The 1D CNN and 1D ResNet shared the top two places in both datasets. In CinC 2017, the 1D ResNet is the best model, followed by the 1D CNN. In CinC 2020, the 1D ResNet surpassed the top-1 solution by a large margin in F1 score and 2% higher specificity. However, these models were still not better than the transformer-based model in sensitivity. Regarding the best model (1D ResNet), the metrics for each class are presented in the Supplementary Table S2. This model worked very well on classifying the class Atrial Fibrillation, Sinus tachycardia, and Left/Right bundle branch block while there are a number of minor patterns that were unable to detect such as Q-wave abnormal or T-wave inversion.

The fivefold cross-validation results, especially the standard deviation of performance metrics, reveals the stability and robustness of the models. Because of lower standard deviation in the cross-validation metrics, the 1D convolutional models are more stable than other models in both datasets, while the performance of gradient-boosting model and Poincaré-based models fluctuate among the splits.

**Figure 4:**
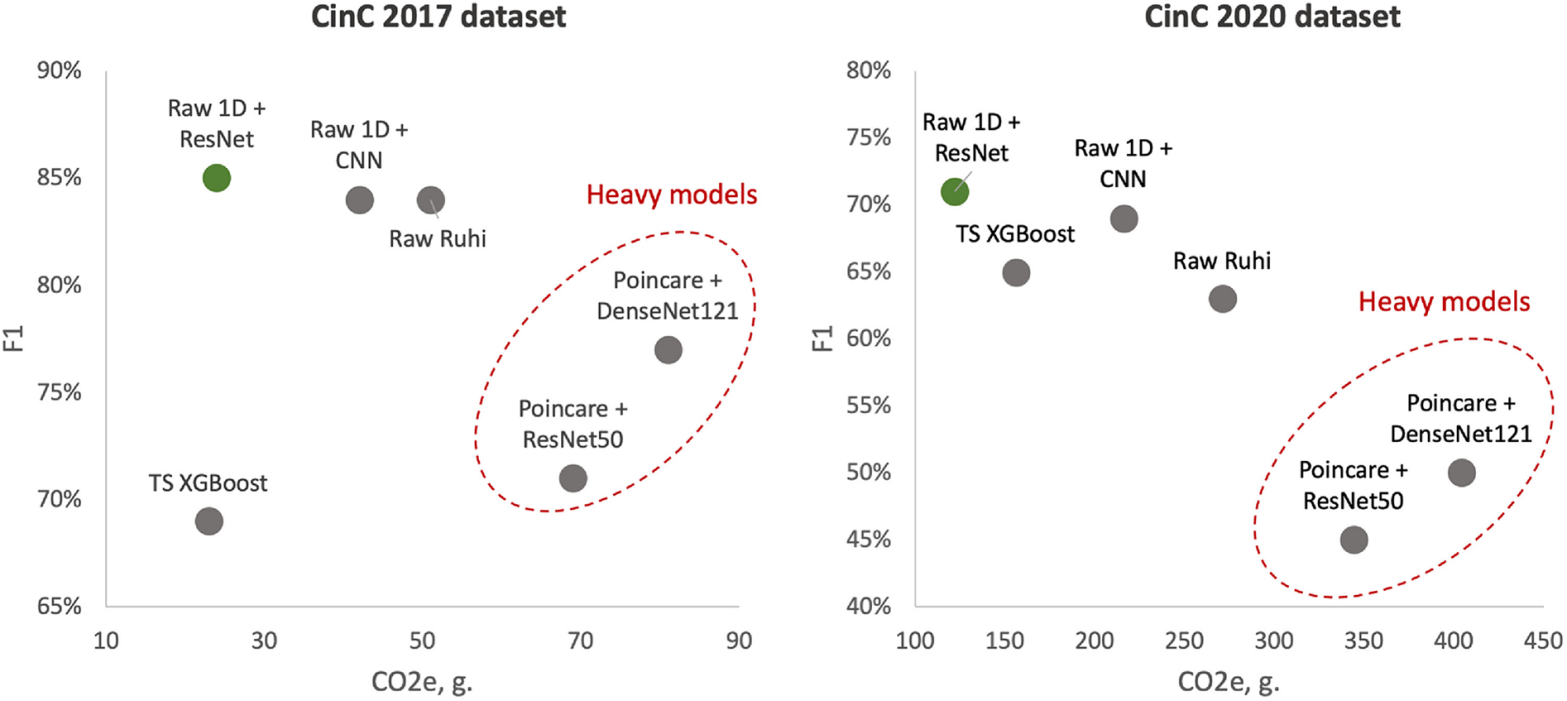
Test F1 score vs. ${\mathrm{CO}}_{2}$ emissions: left side—models learned over CinC 2017 dataset; right side—models learned over CinC 2020 dataset. Dotted red ellipses highlight relatively heavy models.

To better illustrate the tandem “Performance vs. Complexity” for examined models, Figure 4 shows cross-plots on F1 score and ${\mathrm{CO}}_{2}$ emissions for both datasets. In particular, one can reveal that DenseNet121 and ResNet50 models learned over Poincaré diagrams stand out from other models as inefficient while ResNet learned on raw ECG signals outperforms.

We also analyzed the performance of investigated models in each source of the CinC 2020 dataset (Table 4). The ResNet50 was good at the short-term recordings while performing poorly in long-term data. The DenseNet121 was better than ResNet50 in long-term signal classification but did not surpass the 1D Convolutional model. The XGBoost outperformed the others in long-term ECG. However, the number of long-term signals is not very large, so their metrics might not be stable.

**Table 4:** The F1 score on each sources in the CinC 2020 dataset.

Model	G12EC $\bar{l}=10$	PTB-XL $\bar{l}=10$	CPSC $\bar{l}=16$	CPSC-Extra $\bar{l}=16$	PTB $\bar{l}=109$	INCART $\bar{l}=1800$
ResNet50	0.28	0.56	0.35	0.33	0.16	0.20
DenseNet121	0.36	0.58	0.38	0.50	0.77	0.60
1D CNN	0.55	**0.76**	0.61	0.68	0.85	**0.74**
1D ResNet	**0.59**	**0.76**	**0.70**	0.66	0.85	0.70
XGBoost	0.56	0.69	0.55	**0.77**	**0.87**	**0.74**

$\bar{l}$ is the average length of signal in seconds. Bold numbers indicate the performance of the best model.

### 3.2. Efficiency metrics

The results of the efficiency measurement are summarized in Table 5. In terms of power consumption, 2D models are also the most power-hungry models: ResNet50 and DenseNet121 consumed two to three times more energy than the others. On the other hand, the 1D models are more efficient than any other models: the 1D ResNet needed more energy than only XGBoost in CinC 2017 and was the most efficient model in CinC 2020. Despite the 1D CNN having a simpler base block than the 1D ResNet, the CNN required more layers than ResNet (12 CNN blocks vs. 4 ResNet blocks). This made the former require more power while performing less well than the latter.

**Table 5:** Power consumption, equivalent ${\mathrm{CO}}_{2}$ emission, and inference time of each model.

Dataset	Input data	Model	Power (Wh)	${\mathrm{CO}}_{2}$ (g)	Inference time (ms)		
					Processing	Predicting	Total
CinC 2017	Poincaré	ResNet50	127	69	33.7	37.9	71.6
	Poincaré	DenseNet121	148	81	33.7	38.2	71.8
	Raw signal	1D CNN	77	42	**13.5**	25.5	41.0
	Raw signal	1D ResNet	44	24	**13.5**	**18.8**	**32.2**
	Raw signal	Kamaleswaran et al.^a^	92	51	14.3	66.0	80.3
	Time series	XGBoost	**42**	**23**	1717.6	0.2	1717.8
CinC 2020	Poincaré	ResNet50	630	344	227.2	36	263.2
	Poincaré	DenseNet121	740	404	227.2	36.3	263.5
	Raw signal	1D CNN	396	216	**144.3**	29.8	174.1
	Raw signal	1D ResNet	**223**	**122**	**144.3**	**21.6**	**165.9**
	Raw signal	Natarajan et al.^a^	497	271	4.9	833.6	838.5
	Time series	XGBoost	286	156	382.7	0.0	382.7

Bold numbers indicate the performance of the best model.

^a^ The first rank solution.

Equivalent ${\mathrm{CO}}_{2}$ emissions are directly related to the power consumption. Higher power consumption leads to higher ${\mathrm{CO}}_{2}$ emissions. Similar to the power consumption pattern, the DenseNet121 model on the Poincaré input data exhibits the highest ${\mathrm{CO}}_{2}$ emissions for both datasets, while the 1D ResNet model on the raw signal input has the lowest emissions.

Regarding the inference time, although XGBoost had a lightning prediction time, this model dominated the total inference time benchmark, which comes from the heavy processing steps. This problem leads to the fact that XGBoost still inferred 24 times longer than the second place. The Poincaré-based method requires an approximate twofold longer inference time than the 1D CNN or 1D ResNet. This result complies with the mathematical characteristics of the 1D and 2D convolutional operators.

### 3.3. Models interpretation

#### 3.3.1. DenseNet121 on Poincaré diagram classification

**Figure 5:**
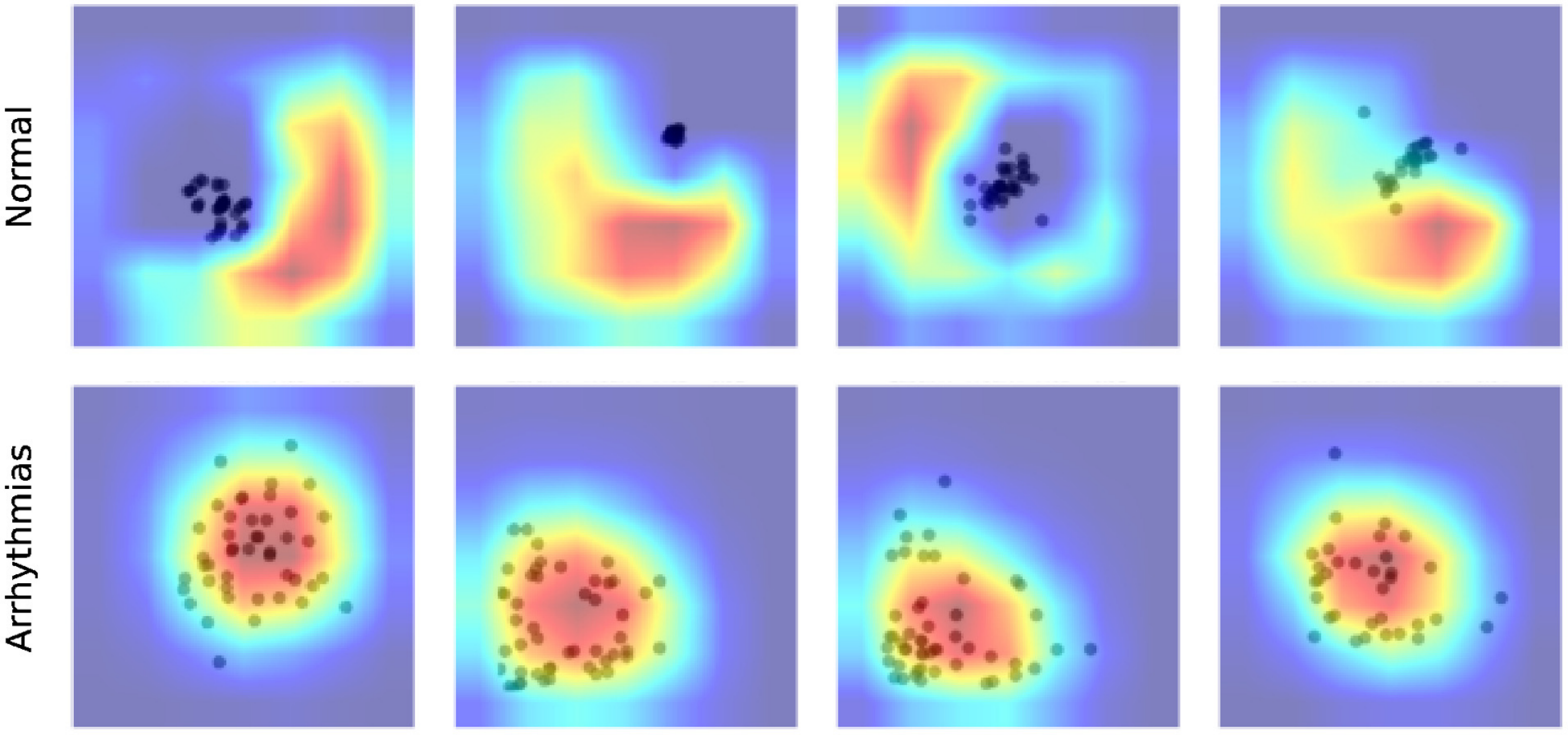
Explaining CinC 2017 predictions on the Poincaré diagrams using GradCAM.

Figure 5 visualized the GradCAM output of DenseNet121 on CinC 2017. We can see how this model processes the Poincaré diagram differently. In the Normal graph, the model focused on the area in the upper left and lower right, while the shape of the point cloud was ignored. In the arrhythmia diagram, the model focuses on the point cloud or the diversion of data.

This behavior of the model is compatible with human knowledge. For ordinary people, we do not expect any data point far away from the diagonal of the diagram. Any data point in the upper left or lower right area is evidence of abnormal changes in heart rate and predicts the problem, whereas in arrhythmia patients, because of the fluctuation in the heartbeat statistics, the data should be very varied and form a spreading cloud in the Poincaré diagram. The bigger cloud shows more variation in heart rate.

#### 3.3.2. 1D ResNet on ECG signal classification

Our work also took advantage of GradCAM to explore the mechanism of the 1D ResNet model. In medical literature, the ECG of Atrial fibrillation was detected by the irregular pattern in P- and T-waves around the QRS complex.

**Figure 6:**
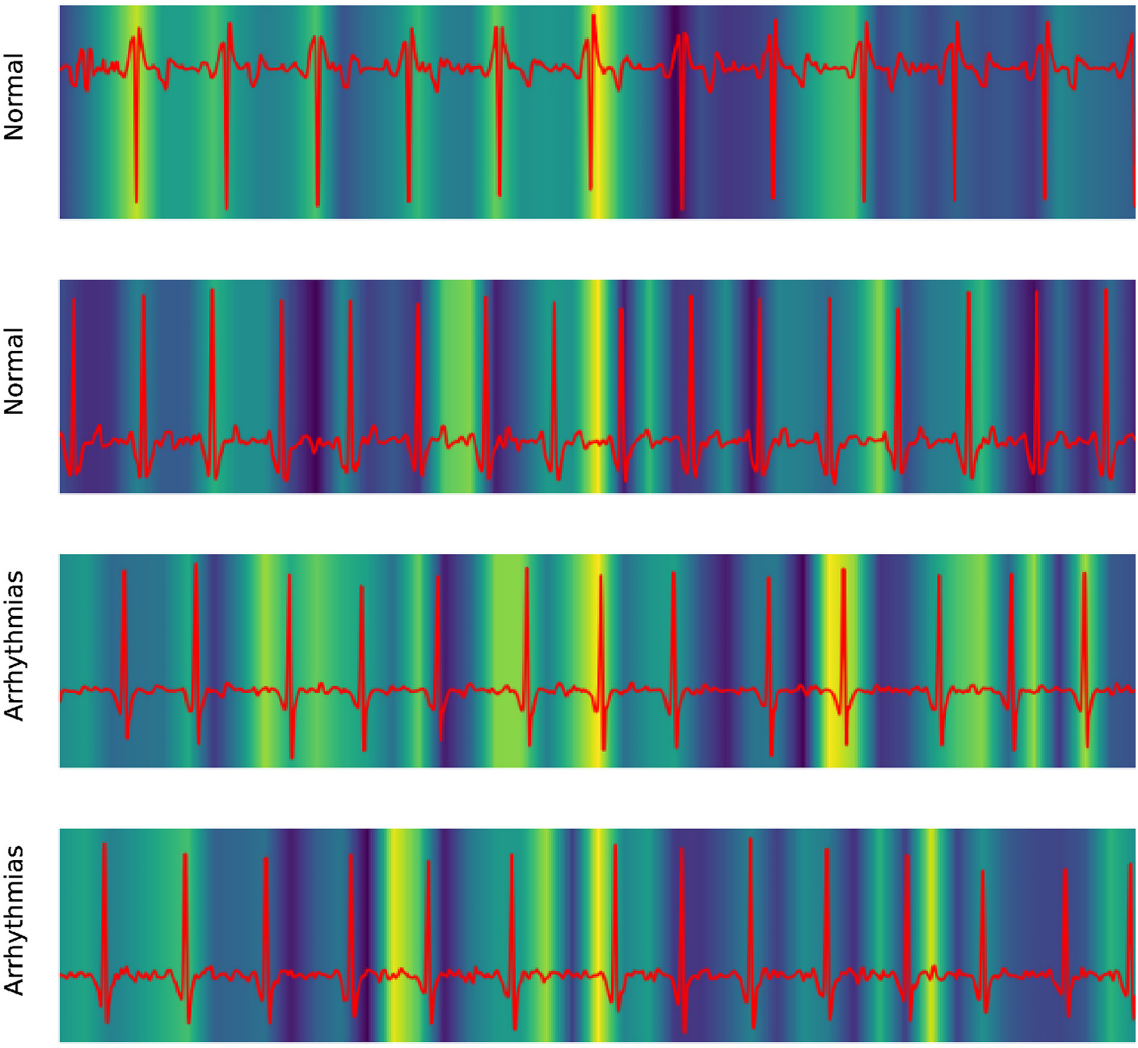
Explaining 1D ResNet decision by GradCAM methods in case of normal regimes and arrhythmia.

Figure 6 shows the focusing points of the 1D ResNet when predicting the AF signal. The yellow area is the segment that the model attracts. These heatmaps show that the classifier focused on the signal at the neighbor of the QRS complex. These regions are corresponding to the P-wave and T-wave of ECG recordings. In fact, the absence or abnormality of P-wave and T-wave is related to the fluctuation of heart rate and predicts arrhythmia disease (22).

#### 3.3.3. Feature importance of XGBoost

To explore how model XGBoost predicts classes, the feature importance score is calculated and summarized in Table 6. The results show that the features relating to the peak of signal, like fft_coefficient and ratio_beyond_r_sigma, are the highly important ones. We can see that the XGBoost model infers the heart rate information indirectly via the peak-related features, after which the model could give the prediction of arrhythmia from heart rate. The detailed description of each feature group could be found in Supplementary Table S3.

**Table 6:** Feature importance score of top 15 feature groups in the model XGBoost.

Group	No. features	Importance score
fft_coefficient	22	0.2138
ratio_beyond_r_sigma	10	0.1989
autocorrelation	8	0.0935
energy_ratio_by_chunks	5	0.0657
index_mass_quantile	5	0.0651
lempel_ziv_complexity	5	0.0483
agg_autocorrelation	3	0.0444
range_count	3	0.0411
spkt_welch_density	3	0.0408
change_quantiles	3	0.0316
quantile	2	0.0279
number_peaks	2	0.0241
count_below	1	0.0185
cwt_coefficients	2	0.0154
number_crossing_m	1	0.0137

### 3.4. Statement on computational resources and environmental impact

This work contributed totally 1.8 kg equivalent ${\mathrm{CO}}_{2}$ emissions when training on a workstation with 1 CPU Intel Core i7-9700F and 1 GPU NVIDIA RTX 3060.

## 4. Discussion

In the study, we have presented various modern approaches to classify cardiac diseases from ECG recordings. The first approach took advantage of the Poincaré diagram and deep learning–based image classifiers. ResNet50 and DenseNet121 architectures were chosen to process the graph. The experimental results figured out that these methods are decent for atrial fibrillation but not good at predicting other types of arrhythmia. In particular, Poincaré-based methods have adequate performance in the CinC 2017 dataset but are not good in the CinC 2020 dataset. The reason is that the information in the Poincaré diagram is limited in NN interval fluctuation, which benefits in predicting arrhythmia but is not much helpful in detecting other cardiovascular disease types. However, NN intervals, and therefore Poincaré diagrams, are much more accessible and can be obtained without the relatively complicated and expensive ECG procedure. Thus, it is still worth studying this approach further.

XGBoost’s performance is more impressive in the subset of long-term than the short-term recordings. This model learned more information in the long signal, and we can transfer that knowledge to short signal via data augmentation. This could be done by segmenting the long signal into many short signals, and then combining to the original dataset before training. Besides that, this gradient-boosting model has a long inference time because of the expensive calculation in the feature engineering step. The feature calculation could be improve by narrowing down the number of calculated features based on the feature importance ranking.

The one-dimensional convolutional model showed the best results in both studied datasets. Especially the 1D ResNet was superior to the first-ranking solution of each challenge. The residual connection showed its advantages in transferring information while keeping the model not too deep. Our experiments proved the superior advantage of the 1D CNN–based model in ECG signal classification over other deep learning architectures. In the experiments on the CinC 2020 dataset, 1D CNN and 1D ResNet were better than the transformer-based model of Natarajan et al., which is well known in sequence tasks. To explain this behavior, we can consider the ECG classification as local pattern recognition, so the model needs to detect the abnormalities in each P, Q, R, S, and T location in the signal. The intensity, appearance, or absence of any component in that complex is evidence of cardiovascular disease. This is also the way the physician reads the ECG recordings. However, the attention mechanism of the transformer architect is likely to collect the global information by connecting the information in many parts of the sequence than focusing on a particular region of the series (23). On the other side, when applied to the sequence processing, the CNN model used the small size filter to focus on a short and fixed segment of signal, which was helpful in capturing the local pattern of each region of the recording.

Although there were papers studying the power consumption on ECG classification problem (24, 25), our paper is the first to investigate the efficiency metrics while training the models, including power consumption, equivalent ${\mathrm{CO}}_{2}$ emissions, as well as the inference time, in a wide range of models. The numerical complexity is also represented by these metrics. Because of the high workload when processing 2D images, 2D ResNet and 2D DenseNet are at the top in power consumption rankings. The XGBoost is energy efficient for the short term, but the power requirement is multiplied many times when training on long-term signals, which came from the complexity of feature engineering. Since the 1D convolution operator is optimized in calculation, unidimensional models like 1D CNN and 1D ResNet are the most energy efficient among the studied methods.

In the aspect of model interpretation, three models (DenseNet, 1D ResNet, and XGBoost) were analyzed to figure out how they discriminate the normal and AF data. The DenseNet detected AF using the heart rate variability, which was measured by the spreading of the data cloud and the presence of data in the upper left and lower right in the Poincaré diagram. On the other hand, the 1D ResNet assessed the AF pattern in raw ECG signal similar to a medical expert: this model focused on the area around the QRS complex, which is also the location of P- and T-waves.

## Data Availability

Our study used the public repository of the challenge CinC 2017 and CinC 2020. The code for reproducing results is available at https://github.com/pnhuy/ecg-analysis. The original contributions presented in the study are included in the article/**Supplementary Material**, further inquiries can be directed to the corresponding author.
