# Supplementary material for: Machine learning-based detection of cardiovascular disease using ECG signals: performance vs. complexity
Source: Front Cardiovasc Med. 2023 Jul 31;10:1229743. doi: 10.3389/fcvm.2023.1229743 (PMC10424727; doi:10.3389/fcvm.2023.1229743)
Supplement: Supplementary file 1 [file Datasheet1.pdf]

Supplementary Material

1 SUPPLEMENTARY TABLES AND FIGURES

Table S1. Description of classes in the training data.

Dataset	Label	Description
CinC 2017	Normal	Normal
	AF	Atrial Fibrillation
	Other	Other Rhythm
	Noisy	Too Noisy to Process
CinC 2020	AF	Atrial fibrillation
	AFL	Atrial flutter
	Brady	Bradycardia
	CRBBB	Complete right bundle branch block
	IAVB	1.00st degree AV block
	IRBBB	Incomplete right bundle branch block
	LAnFB	Left anterior fascicular block
	LAD	Left axis deviation
	LBBB	Left bundle branch block
	LQRSV	Low QRS voltages
	NSIVCB	Nonspecific intraventricular conduction disorder
	PR	Pacing rhythm
	PAC	Premature atrial contraction
	PVC	Premature ventricular contractions
	LPR	Prolonged PR interval
	LQT	Prolonged QT interval
	QAb	Q wave abnormal
	RAD	Right axis deviation
	RBBB	Right bundle branch block
	SA	Sinus arrhythmia
	SB	Sinus bradycardia
	NSR	Normal Sinus rhythm
	STach	Sinus tachycardia
	SVPB	Supraventricular premature beats
	TAb	T wave abnormal
	TInv	T wave inversion
	VPB	Ventricular premature beats

Table S2. Performance metrics of 1D ResNet model on each class.

Dataset	Class	F1	Sensitivity	Specificity
CinC 2017	Normal	0.91	0.94	0.79
	AF	0.83	0.86	0.98
	Other	0.75	0.71	0.95
	Noisy	0.51	0.47	0.99
CinC 2020	AF	0.86	0.86	0.99
	AFL	0.53	0.42	1.00
	Brady	0.26	0.21	1.00
	CRBBB	0.60	0.61	0.99
	IAVB	0.71	0.69	0.98
	IRBBB	0.15	0.09	1.00
	LAD	0.65	0.57	0.97
	LAnFB	0.68	0.65	0.99
	LBBB	0.81	0.82	1.00
	LPR	0.34	0.34	1.00
	LQRSV	0.10	0.06	1.00
	LQT	0.49	0.50	0.98
	NSIVCB	0.11	0.06	1.00
	Other	0.73	0.67	0.83
	PAC	0.50	0.43	0.99
	PR	0.88	0.84	1.00
	PVC	0.04	0.03	1.00
	QAb	0.01	0.00	1.00
	RAD	0.25	0.16	1.00
	RBBB	0.74	0.74	0.99
	SA	0.11	0.06	1.00
	SB	0.72	0.68	0.99
	SNR	0.90	0.91	0.89
	STach	0.83	0.86	0.99
	SVPB	0.00	0.00	1.00
	TAb	0.26	0.18	0.98
	TInv	0.02	0.01	1.00
	VPB	0.22	0.16	1.00

Table S3. Description of feature groups in XGBoost pipeline.

Features Group	Description
`fft_coefficient`	The Fourier coefficients of the one-dimensional discrete Fourier Transform for real input by Fast Fourier transformation algorithm
`ratio_beyond_r_sigma`	Ratio of values that are more than r times standard deviation away from the mean of data.
`autocorrelation`	Calculates the autocorrelation of the specified lag
`energy_ratio_by_chunks`	Calculates the sum of squares of chunk i out of N chunks expressed as a ratio with the sum of squares over the whole series.
`index_mass_quantile`	Calculates the relative index i of time series where $q\%$ of the mass of data lies left of i.
`lempel_ziv_complexity`	Calculate a complexity estimate based on the Lempel-Ziv compression algorithm.
`agg_autocorrelation`	Descriptive statistics on the autocorrelation of the time series.
`range_count`	Count observed values within the interval.
`spkt_welch_density`	This feature calculator estimates the cross power spectral density of the data at different frequencies.
`change_quantiles`	First fixes a corridor given by the quantiles interval of the distribution of data. Then calculates the average, absolute value of consecutive changes of the data inside this corridor.
`quantile`	Calculates a specific quantile of data
`number_peaks`	Calculates the number of peaks of at least support a threshold in data.
`count_below`	Returns the percentage of values in data that are lower than a threshold
`cwt_coefficients`	Calculates a Continuous wavelet transform for the Ricker wavelet
`number_crossing_m`	Calculates the number of crossings of data on a specific threshold
